# Fully Biodegradable Biocomposites with High Chicken Feather Content

**DOI:** 10.3390/polym9110593

**Published:** 2017-11-09

**Authors:** Ibon Aranberri, Sarah Montes, Itxaso Azcune, Alaitz Rekondo, Hans-Jürgen Grande

**Affiliations:** CIDETEC Research Centre, Paseo de Miramón, 196, 20014 Donostia-San Sebastián (Gipuzkoa), Spain; smontes@cidetec.es (S.M.); iazcune@cidetec.es (I.A.); arekondo@cidetec.es (A.R.); hgrande@cidetec.es (H.-J.G.)

**Keywords:** biodegradable biocomposites, thermoplastics, fibres, chicken feathers

## Abstract

The aim of this work was to develop new biodegradable polymeric materials with high loadings of chicken feather (CF). In this study, the effect of CF concentration and the type of biodegradable matrix on the physical, mechanical and thermal properties of the biocomposites was investigated. The selected biopolymers were polylactic acid (PLA), polybutyrate adipate terephthalate (PBAT) and a PLA/thermoplastic copolyester blend. The studied biocomposites were manufactured with a torque rheometer having a CF content of 50 and 60 wt %. Due to the low tensile strength of CFs, the resulting materials were penalized in terms of mechanical properties. However, high-loading CF biocomposites resulted in lightweight and thermal-insulating materials when compared with neat bioplastics. Additionally, the adhesion between CFs and the PLA matrix was also investigated and a significant improvement of the wettability of the feathers was obtained with the alkali treatment of the CFs and the addition of a plasticizer like polyethylene glycol (PEG). Considering all the properties, these 100% fully biodegradable biocomposites could be adequate for panel components, flooring or building materials as an alternative to wood–plastic composites, contributing to the valorisation of chicken feather waste as a renewable material.

## 1. Introduction

There is a growing interest in the research and development of new materials obtained from natural resources as new alternatives to decrease or even replace petroleum-derived plastics. The incorporation of bio-based fillers into polymeric matrices results in a biocomposite [1]. Natural fibres such as flax, hemp and sisal have been widely studied as reinforcements for thermoplastics [2]. Animal fibres such as wool [3], silk [4] and horns [5], which are made of proteins, can also be used as alternative sources of reinforcements and fillers for polymers. All of them represent an opportunity to make the most of renewable and under-utilized raw materials in new application areas.

The poultry industry (including duck, turkey, goose and chicken breeding) generates a huge amount of waste each year. Despite figures regarding feather waste generation varying considerably depending on the source, it is estimated that over 65 million tons of poultry feathers are produced worldwide [6]. According to the European Commission, 13.1 million tons of poultry meat was produced in the European Union (EU-28) only in 2014 [7], with an estimated generation of 3.1 million tons of feather waste. At present, the majority of poultry feathers are disposed of in landfills or incinerated, and a minor part converted into low-nutritional-value animal food. The current solutions do not exploit the opportunity that this proteinaceous material represents, and more importantly, the management of environmental and health concerns as overall waste rises.

CFs, which are composed of 90% keratin, are abundant, cheap, biodegradable [8] and provide an opportunity to replace non-environmentally-friendly raw materials in many applications [9,10,11]. Serine is the most abundant amino acid in the keratin structure and contains a hydroxyl side group, which gives CFs the ability to attract moisture from the air [12]. This particular property makes keratin an effective material for insulation applications [13]. Despite the low mechanical properties of the feathers compared to other natural fibres [2], they have been used, for example, as reinforcement in cement-bonded composites [14], polyurethanes [15], polypropylene (PP)-based composites [16,17] and recycled PP composites [18,19]. Furthermore, a few studies have been performed reinforcing PLA with CFs, obtaining fully biodegradable materials [20,21,22]. Reddy et al. [23] also obtained 100% biodegradable composites using CFs as matrix and jute fibres as reinforcement. When feathers are used as fillers it is important to pay attention to the compatibility with the polymer matrix in order to improve the adhesion with the polymer. According to its amino acid sequence, keratin can be considered to have both hydrophilic and hydrophobic properties, since 39 out of the 95 amino acids in the keratin structure are hydrophilic [24]. Several studies have modified the CF [25] and the polymer matrix [26] for enhancing their compatibility.

Among the aliphatic polyesters, PLA is the most well-known biodegradable polymer and has the greatest commercial potential due to its good aesthetics, mechanical strength, thermal plasticity, biocompatibility and ability to be easily processed with natural fibres [27]. PLA is produced from renewable resources such as corn starch, which is fermented to lactic acid followed by ring-opening or gradual polycondensation polymerization into PLA [28]. The combination of properties of PLA makes it an ideal thermoplastic for a wide array of applications, but it still has some drawbacks when compared to other traditional materials, which are related to its production limitations and brittleness. The latter has a relatively easy solution, since the processability of the biocomposites can be addressed by modifying the PLA with plasticizers such as polyethylene glycol (PEG), citrate ester and glycerol [29]. This is not the case for the former drawback. A huge amount of starting raw material (e.g., corn) is required for PLA production, and having in mind the food category of corn, economic and sustainability issues arise. One approach to reduce the required PLA amount while keeping its benefits is the combination of PLA with natural fibres to create biocomposites.

Among other biodegradable polymers, PBAT is synthesized from fossil fuel-based monomers and shows similar properties to nonbiodegradable polymers like polyethylene. PBAT is flexible and has a higher elongation at break than most biodegradable polyesters, such as PLA, being therefore more suitable for packaging [30].

Bio-Flex^®^ is a commercial product based on polylactic acid and a thermoplastic copolyester, and only a very few articles have published on this system [31].

**Figure 1:**
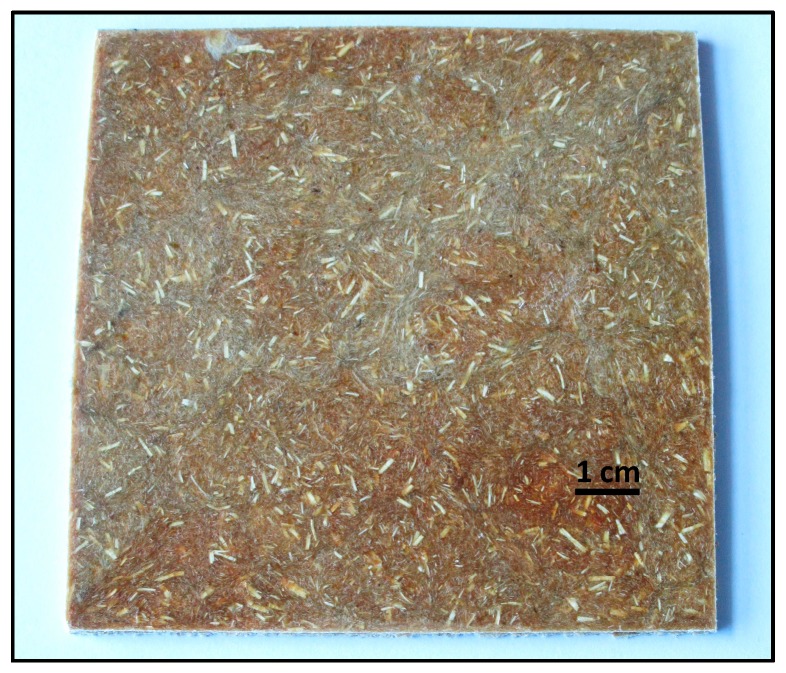
Chicken feather/PLA biocomposite containing 50 wt % of feather.

Even though several studies regarding biocomposites containing CFs up to 20 wt % and PLA have been published, to the best of our knowledge, this is the first example of biocomposites obtained from biodegradable thermoplastics with high loadings of CFs. CF concentrations were selected based on the highest amount of CFs that was possible to incorporate into the polymer matrices, keeping an optimum processability of the blends. Hence, the aim of this study was to develop sustainable biocomposites comprised of 50 and 60 wt % CFs and biodegradable thermoplastic matrices (Figure 1) contributing to the responsible use of materials and zero-waste policies. The selected polymer matrices were PLA, PBAT and a PLA/copolyester blend, which are commercially available, biodegradable and compostable according to EN 13432. Additionally, the effect of the addition of plasticisers to the composite and alkali pre-treatments of the feathers were investigated. The mechanical properties, thermal stability and diffusivity of the resulting biocomposites were studied in order to identify potential applications of these new materials.

## 2. Materials and Methods

### 2.1. Materials

**Figure 2:**
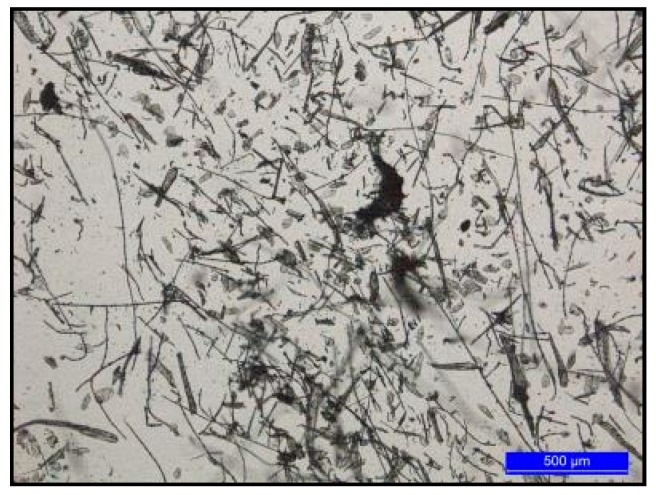
Optical image of ground chicken feathers.

Sanitized CFs from Grupo SADA (Madrid, Spain) were ground in a universal cutting mill Pulverisette 19 (Fritsch, Germany) at a rotor rotational speed of 2800 rpm and at a sieve insert size of 1 mm. According to the optical microscope images (Figure 2), ground feathers showed a wide size distribution ranging from 100 μm to a few mm. The different polymers used in this work are detailed in Table 1. According to the EN 13432, all the matrices are biodegradable and compostable. Sodium hydroxide (NaOH, 97%) and polyethylene glycol with molecular weight of 400 g/mol (PEG 400) were purchased from Aldrich (St. Louis, MO, USA).

### 2.2. Alkali Treatment of the CFs

CFs were immersed in NaOH solution (5% *w*/*v*) for 2 h at room temperature. The feathers were then washed with distilled water until the rinse water no longer indicated any alkalinity [32]. After washing, the feathers were kept in an oven at 80 °C for 6 h.

### 2.3. Biocomposite Preparation

The biocomposites were manufactured using a torque rheometer HAAKE PolyLab QC (Karlsruhe, Germany). Prior to blending, ground CFs and the polymers were dried at 80 °C for 6 h. The contents of CF in the biocomposites were 50 and 60 wt %. ING, ECO, BIO biopolymers were first melted at 170, 140 and 170 °C, respectively, and CFs were added later while mixing for 3 min. For the ING/PEG400 blends, 10 wt % of PEG400 was mixed before the CFs were incorporated. The blends were compression-molded in a Vogt 600T laboratory hot press machine (Maschinen + Technik Vogt GmbH, Möhnesee, Germany) into sheets of 90 × 90 × 2.1 mm^3^. Consolidation was carried out at 200 °C for 3 min and finally, the square plates were cooled with air. TGA analyses of the biocomposites were performed previously in order to set the consolidation conditions.

### 2.4. Characterization of the Biocomposites

#### 2.4.1. Density of the Biocomposites

The density of the biocomposites was determined experimentally according to the ISO 9427 [33]. Three rectangular samples of each composite with a known volume were weighed and the density was determined as the ratio of the mass to volume. The average and standard deviation were reported.

#### 2.4.2. Water Absorption of Biocomposites

Water absorption of the biocomposites was determined by immersion of the specimens vertically in distilled water at 25 °C for 24 h (ASTM D570-98) [34]. First, rectangular specimens (24 mm × 12 mm × 2.1 mm) were cut from tensile testing fracture specimens and air-dried at 60 °C for 24 h, cooled in a desiccator and weighed (conditioned weight). Then, samples of the biocomposites were soaked in water for 48 h and wiped with paper to remove the excess of water on the surface of the specimens before weighing (wet weight) at fixed time intervals. Three specimens were tested with an analytical balance of 0.1 mg precision and the average and standard deviation were reported. The percentage of water absorption (*WA* in %) was calculated using Equation (1):

(1) $$WA\;\left(\%\right)=\frac{\mathrm{wet}\;\mathrm{weight}-\mathrm{conditioned}\;\mathrm{weight}}{\mathrm{conditioned}\;\mathrm{weight}}\times 100$$

#### 2.4.3. Thermogravimetric Analysis (TGA)

The thermal stability was measured by thermogravimetric analysis using a TGA Q500 (TA Instruments, New Castle, DE, USA). Dynamic measurements were performed from 25 to 600 °C at a heating rate of 10 °C/min by using constant nitrogen flow of 60 mL/min to prevent thermal oxidation processes of the polymer sample. The temperatures at 5%, 10%, 25% and 50% of weight loss were calculated. Sample weight was approximately 10 mg.

#### 2.4.4. Dynamic Scanning Calorimetry (DSC)

Thermal properties of CFs, ING, BIO, ECO and the biocomposites containing 50 and 60 wt % CFs were determined with a Discovery DSC 25 auto (TA Instruments, New Castle, DE, USA) at a scan rate of 10 °C·min^−1^ over the temperature range 25–200 °C for CF and ING series and −70–200 °C for ECO and BIO series due to their differences in thermal properties. The measurements were carried out using 5.00 ± 0.50 mg samples under a nitrogen atmosphere (50 mL·min^−1^). All of the thermal properties were obtained from the second heating curves in order to evaluate the effect of the filler, as nucleating agent, during the processing. The degree of crystallinity (χ_c_) was calculated from the following Equation (2):

(2) $$\chi_{c}\left(\%\right)=\frac{100}{w}\times \frac{\left|\Delta H_{m}+\Delta H_{\mathrm{cc}}\right|}{\Delta H_{m}^{o}},$$

where $\Delta H_{m}$ and $\Delta H_{\mathrm{cc}}$ are the enthalpy of fusion and cold-crystallization at melting and crystallization temperatures, respectively, *w* is the weight fraction of neat polymer in the sample and $\Delta H_{m}^{0}$ is the melting enthalpy of the 100% crystalline polymer. $\Delta H_{m}^{0}$ was taken as 93.7 and 114 J/g for PLA and PBAT, respectively. For the BIO series, $\Delta H_{m}^{0\;}$ was also taken as 93.7 J/g after the assumption that Bio-Flex 6611 is mainly based on PLA, and no other melting peak was observed in the analysed range.

#### 2.4.5. Mechanical Testing

The tensile properties of the biocomposites (Young’s modulus, tensile strength, and elongation at break) were evaluated using a tensile test according to the ISO 527 [35] standard with a universal testing machine model 3365 (Instron, Norwood, MA, USA) and controlled by Bluehill Lite software developed by Instron (Norwood, MA, USA). The initial length of the test specimens was 25.4 mm and a cross-head speed of 10 mm/min was used. The number of tested specimens for the mechanical properties was 5 for average calculations.

#### 2.4.6. Thermal Diffusivity

The thermal properties were measured utilizing a light flash analyzer (LFA, Nanoflash 447 by Netzsch, Ahlden, Germany) following the ASTM E1461 [36]. This analyser operated a xenon flash light that induced a pulse of energy on one side of the sample. Such a pulse increased the sample temperature and an indium antimonide (InSb) infrared detector measured the temperature response time to the pulse of energy on the other side of the sample. The response time was used to calculate the thermal diffusivity.

#### 2.4.7. FE–SEM

The CF/polymer interface was analysed by scanning electron microscopy of the fractured surface of the composites. The microphotographs were taken with a Carl Zeiss Ultra Plus field-emission–scanning electron microscope (FE–SEM, Oberkochen, Germany) equipped with an energy dispersive X-ray spectrometer (EDXS). Prior to FE–SEM analysis, samples were Au-coated.

## 3. Results and Discussions

### 3.1. Density

One of the main reasons of using biocomposites containing a high concentration of CF is the high-density reduction that is expected due to the low density of these feathers. Compared to other natural fibres like wool 1.31 g/cm^3^, jute 1.3 g/cm^3^ and coir 1.2 g/cm^3^, the density of the chicken feather fibres ranges between 0.8 g/cm^3^ [37] and 0.9 g/cm^3^ [38]. This variation may be due to the different source of feathers. Hence, chicken feather inclusion in a thermoplastic matrix could potentially lower biocomposite density more than any other reinforcing natural fibre.

In Table 2 are summarised density values and the percent reduction of the biocomposites compared to the neat polymers. The density values determined for the processed ING, ECO and BIO matrices are 1.16, 1.22 and 1.28 g/cm^3^, respectively. As expected, the addition of CFs decreased the density values of all the biocomposites, as has been reported by other researchers [39]. The higher density of the neat matrix (ECO and BIO), the more pronounced was the reduction of the composite material. The addition of 50 wt % of CF led to 13.11% and 15.63% density reductions, whereas the addition of 60 wt % of CF lead to 17.21% and 20.31%, respectively.

The reduction is attributed to the high void volume of the biocomposites due to the presence of the hollow structure of the CFs [40] which were preserved after the compression molding conditions.

### 3.2. Water Absorption

**Figure 3:**
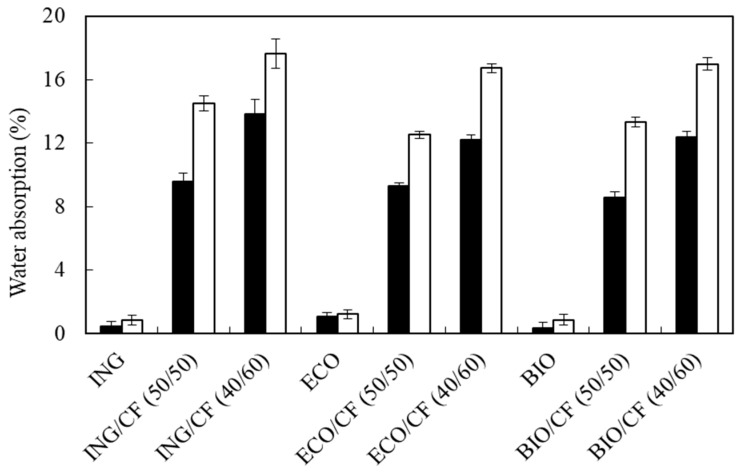
Water absorption of the polymers and biocomposites after 24 h (black) and 48 h (white) of immersion in water at 25 °C.

In order to study the dimensional stability of the biocomposites, all samples were immersed in water at 25 °C for 48 h. Figure 3 shows the water gain after 24 and 48 h of immersion of water. The water absorption of the composite samples was calculated with (1). The water gain in the neat polymers after 24 h was 0.46%, 1.1% and 0.32% for ING, BIO and ECO, respectively. These low values were expected since the polymeric matrices are hydrophobic. After 48 h of immersion, all the composite samples gained water in the range between 12.5–14.5% for 50 wt % and 16.6–17.6% for 60 wt % of CF. As can been seen, the mass uptake of the biocomposites increased as the CF loading and the immersion time increased. The water gain of the composites is mainly attributed to the hydrophilic polymer backbone of the CF [41]. ING-based composites showed the highest water absorption. This effect is probably due to neat PLA samples containing more polar groups in the polymer chain than the other polymer matrices. These results are in agreement with the results published by Carrillo et al., in which the addition of CFs promotes water absorption in biocomposites based on PLA [20,39] and polyolefins [39].

### 3.3. Thermogravimetric Analysis (TGA)

**Figure 4:**
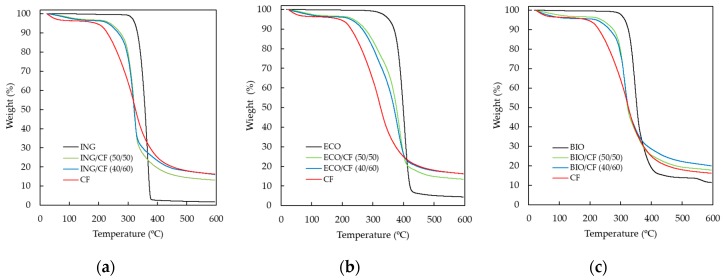
TGA curves of (**a**) ING-based biocomposites; (**b**) ECO-based biocomposites and (**c**) BIO-based biocomposites.

The thermal stability of neat polymers and polymer/CF biocomposites was investigated with TGA. The TGA curves presented in Figure 4a–c show thermal decomposition of CFs, neat polymers (ING, ECO and BIO) and the CF-containing biocomposites. The weight loss of the CFs is represented in the three figures as reference. Three weight-loss steps can be seen in the case of CFs. The weight loss in the first stage, from 50 to 250 °C, is due to the evaporation of absorbed water from the hydrophilic groups of the CFs; the second step, with a higher rate, between 250 and 400 °C, undergoes degradation associated with the destruction of disulphide bonds and the elimination of H_2_S originating from amino acid cysteine [42]; and from 400 °C onwards, the keratin partially decomposes. According to Figure 4a–c, the biocomposites also degrade through three stages and the degradation starting temperature depends on the type of matrix. The degradation starting temperature of the ING and the corresponding biocomposites is slightly lower than that of ECO and BIO and their biocomposites. Moreover, the degradation rate is faster for ING-based biocomposites. All biocomposites are thermally stable until 230–240 °C; at this temperature, composites begin thermal degradation due to presence of CFs also in three stages. For temperatures above 400 °C, all the polymer matrices were completely decomposed and an inorganic residue coming from the CFs was left behind after the decompositions of the CF-containing composites. Composites containing 60 wt % CF started degrading at lower temperatures than composites containing 50 wt % CF due to the higher keratin content. These results are in agreement with previous studies of PLA/CF biocomposites containing up to 10 wt % [21] and 30 wt % of CFs [25].

In Table 3, the 5%, 25% and 50% weight-loss temperatures are listed for all the specimens shown in Figure 4a–c.

### 3.4. DSC Analysis

**Figure 5:**
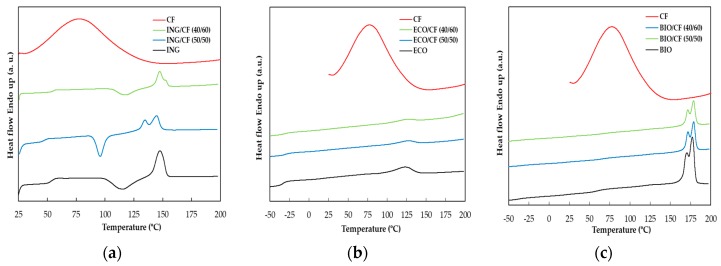
DSC curves of (**a**) ING-based biocomposites; (**b**) ECO-based biocomposites and (**c**) BIO-based biocomposites.

In order to study the effect of the CFs on the thermal properties of the neat polymers, DSC analysis of CFs, neat polymers and the corresponding biocomposites was performed. For CFs (shown in Figure 5a–c as reference), a large low-temperature endothermic peak was observed at 77 °C. This peak ranges from 30 to 130 °C approximately and shows the amount of bound water in the keratin structure, and on occasion is referred as the “denaturation” temperature. Figure 5a shows the DSC curves of CF/ING biocomposites. The glass transition temperature decreased from 53.6 to 47.4 °C and increased to 55.6 °C with addition of 50 and 60 wt % of CF, respectively. This lack of trend of the *T*_g_ of PLA may be due to: (i) heterogeneity of the material. The high concentration of CF may contribute to a poor mixture of both phases; (ii) the DSC thermogram of the CFs may affect the output of the *T*_g_ values of the biocomposites; and (iii) high disparity in the thermal properties of the different part of the feathers [43].

For the ECO series shown in Figure 5b, the *T*_g_ increased and the Δ*H*_m_, *T*_m_ and the degree of the crystallinity of the biocomposites decreased in the presence of CFs. In this series, since the melting temperatures of the samples were between 116.8 and 120.4 °C, the DSC curve of the CFs in this range overlaps with the DSC curves of the ECO series, affecting the measurements of the melting enthalpy.

In Figure 5c the DSC curves of the BIO series are shown. All the samples based on a copolymer that contains PLA showed two *T*_g_, both increasing significantly after addition of 50 and 60 wt % of CFs. The melting temperatures *T*_m_ increased slightly and Δ*H*_m_ decreased more than 50%. The crystallinity of PLA in the BIO series increased from 30.5% to 36.0% when the biocomposite was reinforced with 60 wt % of CFs, observing the same effect as observed in the ING series. According to [44,45], there are two main factors affecting the crystallinity of polymer composites: (i) additives having a nucleating effect that results in an increased crystallinity, and (ii) additives hindering the migration and diffusion of polymer chains to the surface of the growing polymer crystal, lowering crystallinity.

The crystallinity was found to increase in the ING and BIO series, both containing PLA, in agreement with Cheng [20], who reported that chicken feather fibres play the role having a nucleating effect in PLA. In the same study, it was observed that the crystallization peaks became narrower with the addition of up to 10 wt % of CFs, indicating an increase of the crystallization rate. A similar effect was observed for the ING/CF (50/50) biocomposite. However, a broader crystallization peak was found for the ING/CF (40/60), probably due to a more heterogeneous crystallization process, which can be attributed to a higher CF loading. Regarding the ECO-based biocomposites, the crystallinity decreased. In this case, the addition of CFs may disrupt the crystallite formation of PBAT due to the increasing of viscosity, hence leading to less-ordered and smaller crystals. Detailed results of the thermal properties obtained from DSC are summarized in Table 4.

### 3.5. Mechanical Properties of the Biocomposites

Previous work by Cheung [46] proved that the mechanical properties of chicken feather fibre/PLA biocomposites were determined by the feather loading. As a matter of fact, the tensile strength of PLA decreases with increasing the content of CF, which is ascribed to the fact that the strength of CF is insufficient. Cheng et al. [21] investigated 2, 5, 8 and 10 wt % feather concentration and showed that even though the tensile properties of the composites were improved with 2 and 5 wt % loading compared to the neat PLA, increasing feather amount resulted in a significant worsening of the mechanical properties. Therefore, it was expected that 50 and 60 wt % of feather content could decrease the mechanical properties considerably. The three polymer matrices that have been investigated in this work have different initial mechanical properties and thus have shown different performance upon the addition of CFs.

Under some conditions, alkali treatment could significantly improve adhesion at the interface and contribute to penetrate the fibre into the polymer matrix. Alkali treatment’s main purpose is to remove some wax and oils, thereby increasing surface roughness and reducing its hydrophilic nature [47].

**Figure 6:**
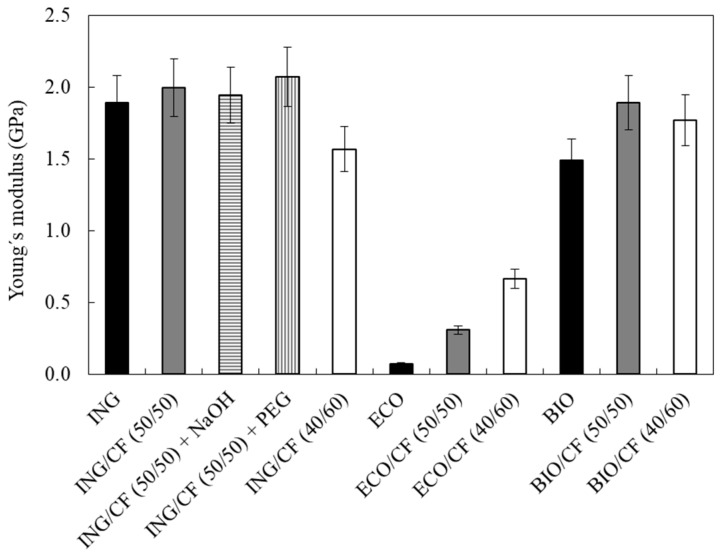
Young’s moduli values of neat polymers and the different biocomposites containing 50 and 60 wt % of CFs.

**Figure 7:**
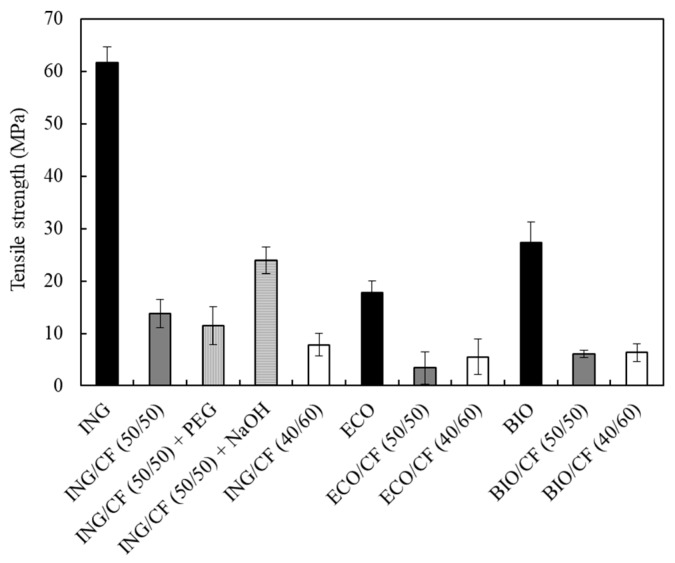
Tensile strength values of neat polymers and the different biocomposites containing 50 and 60 wt % of CFs.

**Figure 8:**
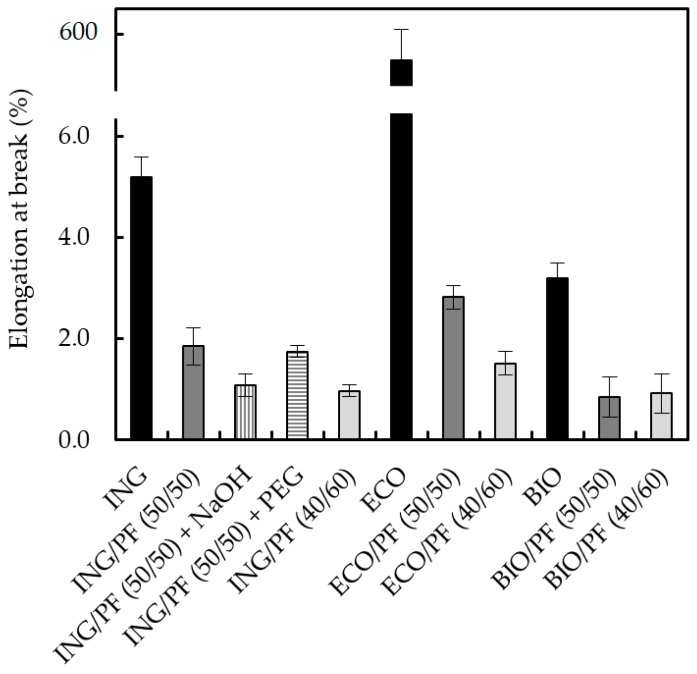
Elongation-at-break values of neat polymers and the different biocomposites containing 50 and 60 wt % of CFs.

Figure 6, Figure 7 and Figure 8 show how three main variables affect the mechanical properties (Young’s modulus, tensile strength and elongation at break) of the biocomposites: (1) the type of the polymer matrix, (2) the CF content and (3) the alkali treatment of the feathers and blending of ING with PEG.

In Figure 6, the Young’s moduli of the biocomposites are presented. This parameter measures the stiffness of the materials. ING and BIO showed a similar trend upon the addition of CFs.

For biocomposites based on ING, the addition of 50 wt % of CFs has no major effect on the Young’s modulus compared to the neat polymer. Even when alkali-treated CFs and PEG as plasticiser were added, only a minor increase was observed, reaching a maximum value of 2.1 GPa. However, increasing the feather loading, that is, the ING/CF (40/60) biocomposite, showed a pronounced decrease of Young’s modulus of 20%.

For ECO/CF biocomposites, the reference value of the neat polymer is lower than that of ING and BIO and hence, the effect of reinforcing ECO with CF is substantial. The Young’s moduli are 70, 300 and 670 MPa for 0, 50 and 60 wt % of CFs, respectively, with an increase up to 1000%. For BIO/CF biocomposites, adding feathers has a positive effect, but as observed in the first case, the increase of CF loading to 60 wt % is detrimental. The differences observed on the trends of the mechanical properties of the biocomposites are probably due to the different degrees of compatibility between the matrices and the feathers.

In Figure 7, the tensile strengths of the biocomposites are shown. This property is a measurement of the force required to break the material per unit area. For all the series, the effect on the tensile strength was similar, decreasing when 50 and 60 wt % of CF was added. This effect is probably a combination of the high CF content and hence poor mixing between the components, and low adhesion between the matrices and the fibres [43]. For ING/CF specimens, the alkali treatment of the CFs increased the tensile strength, with respect to the composite with untreated CFs, from 13.7 to 24 MPa.

Figure 8 shows the elongation at break of the different samples. The elongation at break, which is the ratio between changed length and initial length after breakage, showed a dramatic decrease with CFs obtaining less ductile materials. The most obvious case is the ECO/CF series, varying the elongation at break from 570% for pure ECO to 2.5% for ECO/CF (50/50). When the percentage of the CF loading was increased in this series, the elongation at break further decreased. For all the series, when the percentage of CF was 50 wt %, the ductility of the biocomposites decreased, which indicates that the CFs had hardened the biocomposites and reduced their ductility. For the ING series, no clear effect of the alkali treatment or PEG was observed. When the CF loading was increased to 60 wt %, the elongation at break of ING/CF (40/60) and ECO/CF (40/60) was reduced. The elongation at break of ING/CF (40/60) was not further reduced.

### 3.6. Morphology of the Biocomposites

**Figure 9:**
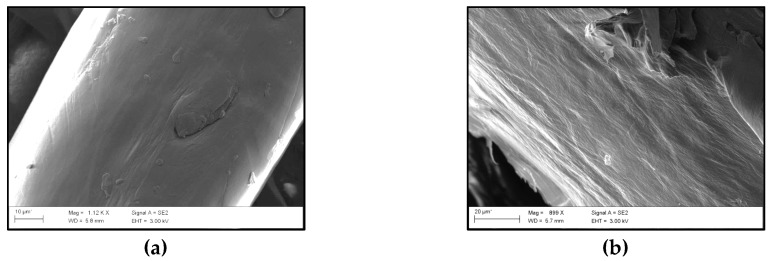

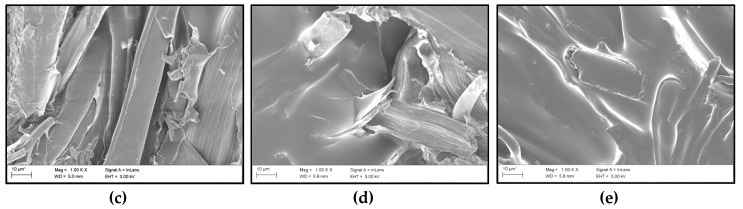
FE–SEM images of (**a**) untreated CFs; (**b**) NaOH-treated CFs; (**c**) fractured ING/CF (50/50); (**d**) fractured ING/NaOH–CF (50/50) and (**e**) fractured PEG–ING/CF (50/50).

The effects of the two different treatments on the adhesion between the PLA matrix and CFs were investigated by FE–SEM. Micrographs of raw CFs and NaOH-treated CFs are shown in Figure 9a,b, respectively. As can be observed, alkali treatment increases the roughness of the CFs, increasing the surface area of the fibres available for contact to the matrix. Bismarck et al. [48] also observed this effect in natural fibres. Additionally, alkali treatment also removes hydrophilic components located on the fibre surface [47]. The micrographs of the fractured surface of the untreated CF/ING biocomposites can be seen in Figure 9c. This figure shows individual CFs, which indicates that the fibres have been not mixed well with the matrix due to a low adhesion between the matrix and the fibres. As can be seen in Figure 9d, the ING/NaOH–CF biocomposites show a good dispersion of the NaOH–CFs in the ING matrix compared to the appearance of the ING/CF biocomposites (Figure 9c), enhancing the CF wettability in the polymer matrix and improving the tensile strength of the biocomposites. In the case of the biocomposites containing the PEG, see Figure 9e; chicken fibres were completely covered by the polymer, however, no improvement in the tensile strength and modulus was observed.

### 3.7. Thermal Diffusivity

**Figure 10:**
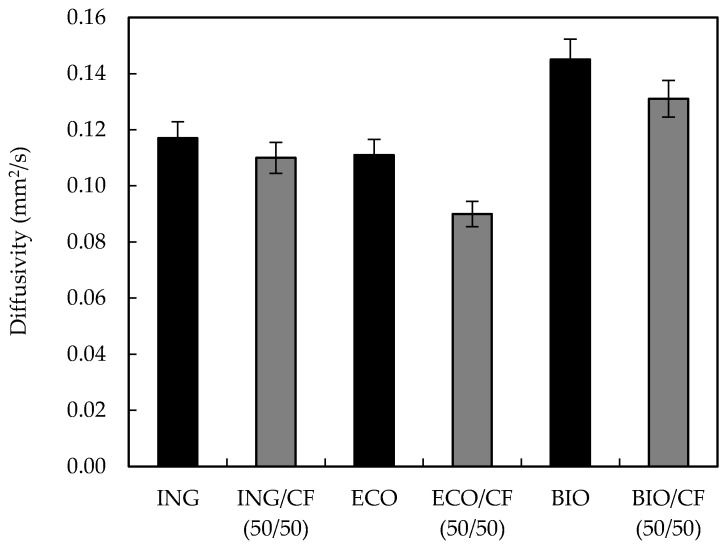
Diffusivity values of neat polymers and polymers reinforced with 50 wt % CFs.

Thermal diffusivity measures the rate at which heat flows through a material. It is a measure of the rate at which a body with a non-uniform temperature reaches a state of thermal equilibrium. The measured thermal diffusivity of the biocomposites at 25 °C and containing 50 wt % of CFs are shown in Figure 10. It can be seen that when the CFs were added, the thermal diffusivity of the biocomposites was reduced. The thermal diffusivity reduction varied from 5% for ING and 9.5% for BIO to 18.9% for ECO. The CFs showed a hollow honeycomb-shaped structure, which acted as air and heat insulators causing a decrease of thermal diffusivity of the CF-containing biocomposites [40]. These results mean that biocomposites containing CFs will require longer time to be heated or cooled than the unfilled polymer, indicating that the CF-reinforced biocomposites show improved thermal insulation properties. Qin, X. [13] reported a similar trend when PLA sheets were reinforced with CF mats.

## 4. Conclusions

In this work, fully biodegradable biocomposites with 50 and 60 wt % of CFs were successfully developed and their density, water absorption, thermal stability, mechanical properties and thermal diffusivity were investigated.

CF/polymer biocomposites with high content of CF showed a decrease in density values unlike any other natural or synthetic fibres, forming lightweight fully biodegradable materials. Regarding water absorption, the biocomposites gained from 12% to 17% of weight after 48 h of immersion, due to the hydrophilic amino acid groups found in the structure of the feathers.

The thermogravimetric curves revealed that the thermal stability of the biocomposites is lowered by the addition of feathers, due to their high content of thermally unstable keratin.

Mechanical properties of the CF-containing biocomposites showed lower tensile strength and lower elongation-at-break values compared to those of neat polymers, whereas the elastic moduli depended on the matrix type. The elastic moduli of the biocomposites were increased for ECO- and BIO-based biocomposites due to the low values of the neat polymers. In these cases, the biocomposites became stiffer. In the ING series, the moduli were not affected by the addition of the CFs for the ING/CF (50/50) biocomposite.

The presence of honeycomb structures in CFs provides air- and heat-insulating capabilities to the biocomposites, lowering the thermal diffusivity of the biocomposites. The results showed that the CFs could be considered as isolator components in buildings to reduce heat transfer and hence decrease energy consumption.

Taking into account the overall properties observed, these materials could be used as an alternative to wood–plastic composites in medium-density fibreboards (MDF) composites [49], decking materials and many other applications [50], in which low density, low thermal diffusivity and low water absorption are also required.

This approach would contribute to a more efficient use of natural resources and take advantage of this material that is produced in huge amounts and is currently underutilized by the poultry industry. It is intended that the use of 100% biodegradable materials will contribute to sustainability and a reduction in the environmental impact associated with the disposal of non-biodegradable polymers.

## Figures and Tables

**Table 1:** Properties of the biopolymers obtained from their technical datasheets.

Polymer (Sample Code)	Type of Matrix	Melting Temperature (°C)	Density (g/cm^3^)	Supplier
Ingeo 2003D (ING)	PLA	145–160	1.24	NatureWorks (Minnetonka, MN, USA)
Ecoflex C1200 (ECO)	PBAT	120	1.25–1.27	BASF (Ludwigshafen, Germany)
Bio-Flex 6611 (BIO)	PLA/copolyester blend	150–170	1.29	FKuR (Willich, Germany)

**Table 2:** Density values and percentage of density reduction of the biocomposites compared to neat polymer.

Matrix	Polymer/CF Ratio	Density (g/cm^3^)	% of Density Reduction
			Compared to Neat Polymer
ING	100/0	1.16	-
	50/50	1.10	5.17
	40/60	1.00	13.79
ECO	100/0	1.22	-
	50/50	1.06	13.11
	40/60	1.01	17.21
BIO	100/0	1.28	-
	50/50	1.08	15.63
	4960	1.02	20.31

**Table 3:** Thermal characterization of the neat polymers and biocomposites.

Samples	*T* (5%) (°C)	*T* (25%) (°C)	*T* (50%) (°C)
CF	191	276	325
ING	323	345	358
ING/CF (50/50)	238	305	322
ING/CF (40/60)	231	302	321
ECO	352	386	398
ECO/CF (50/50)	236	330	377
ECO/CF (40/60)	227	316	369
BIO	314	339	353
BIO/CF (50/50)	239	304	323
BIO/CF (40/60)	221	303	325

**Table 4:** Thermal properties of ING, ECO, BIO and their corresponding biocomposites.

Sample	*T*_g1_ (°C)	*T*_g2_ (°C)	Δ*H*cc (J/g)	*T*_m_ (°C)	Δ*H*_m_ (J/g)	χ (%)
ING		53.6	19.5	147.6	22.7	3.48
ING/CF (50/50)		47.4	15.2	144.6	17.9	5.78
ING/CF (40/60)		55.6	9.00	147.4	11.8	7.50
ECO	−34.1			120.4	9.8	8.60
ECO/CF (50/50)	−30.4			116.8	4.3	7.54
ECO/CF (40/60)	−30.7			116.6	2.7	5.92
BIO	−37.8	58.7		176.9	28.6	30.5
BIO/CF (50/50)	−33.1	65.6		178.7	14.4	30.7
BIO/CF (40/60)	−32.1	64.8		178.5	13.5	36.0
